# Polystyrene Nanoplastics Elicit Multiple Responses in Immune Cells of the *Eisenia fetida* (*Savigny*, 1826)

**DOI:** 10.3390/toxics13010018

**Published:** 2024-12-26

**Authors:** Huijian Shi, Yaoyue Wang, Xiangxiang Li, Xiaoyang Wang, Yuntao Qi, Shaoyang Hu, Rutao Liu

**Affiliations:** Jinan Ecological and Environmental Monitoring Center, Jinan 250104, China; 13791090303@163.com (H.S.); wangyaoyue2021@163.com (Y.W.); lixiangx2023@163.com (X.L.); xxwww2020@sina.com (X.W.); 202232932@mail.sdu.edu.cn (Y.Q.); hushaoyang@sdu.edu.cn (S.H.)

**Keywords:** NPs, oxidative stress, immunity, apoptosis

## Abstract

The improper disposal of plastic products/wastes can lead to the release of nanoplastics (NPs) into environmental media, especially soil. Nevertheless, their toxicity mechanisms in soil invertebrates remain unclear. This study investigated the impact of polystyrene NPs on *Eisenia fetida* (*Savigny*, 1826) immune cells, focusing on oxidative stress, immune responses, apoptosis, and necrosis. Results showed that 100 nm NPs were internalized into the cells, causing cytotoxicity. NPs were observed to inhibit cell viability by increasing reactive oxygen species, decreasing the levels of antioxidants (e.g., superoxide dismutase, catalase, and glutathione), and inducing lipid peroxidation and DNA oxidation. Additionally, assays on neutral red retention time, lysozyme activity, and Ca^2^⁺ levels demonstrated that NPs resulted in a loss of lysosomal membrane stability and a reduction in immune resistance. The depolarization of the mitochondrial membrane potential and the results of the apoptosis assays confirmed that the NPs induced the onset of early apoptosis. The difficulty of the NP in causing cell death by disrupting the plasma membrane was demonstrated by the results of the lactate dehydrogenase release assays in relation to cell necrosis. This research provides cellular-level insights into the ecological risks of NP exposure on soil fauna.

## 1. Introduction

In the 20th century, the consumption of plastics exploded due to the needs of industry, agriculture, construction, and other fields, as well as the daily lives of human beings. In the 1950s, the total global production of plastics was around 1.5 million tons [1], while, in 2022, the production had reached over 400.3 million tons [2]. Statistics show that, between 1950 and 2015, only 21% of plastic waste was recycled or incinerated, with the majority of the rest being landfilled or released into the environment [1]. It is projected that the quantity of plastic waste present in natural environments will exceed 120 million tons by the year 2050 [3]. In the presence of sunlight, seawater, wind, and various organisms, these plastics tend to decompose or degrade, forming new pollutants such as microplastics (MPs, ≤5 mm), nanoplastics (NPs, less than 100 nm), and a variety of complex composite pollutants [4,5]. Currently, MPs have been identified in environmental media such as oceans, rivers, soil, and atmosphere [6]. Of these, the soil environment has been demonstrated to act as both a significant sink and source of MP pollution [7,8].

M/NP has been demonstrated to exert a range of toxic effects, including oxidative stress, inflammatory responses, immunotoxicity, neurotoxicity, and growth and reproductive impairments [9,10,11]. However, the majority of these reports are based on studies conducted in aquatic systems [12,13]. This may be attributed to the fact that M/NP is more dynamic in aquatic environments. It is estimated that soil contains between 1.5 and 6.6 tons of MP, which is one to two orders of magnitude more than the ocean surface [14]. The soil is a complex porous medium, and MP particles in soil can migrate and disperse through processes such as soil erosion, surface runoff, and agricultural activities to reach deeper soil layers or other environmental systems [15]. A considerable body of evidence attests to the fact that the input of M/NP into soil systems can result in alterations to soil properties and the occurrence of adverse effects on soil microorganisms, plants, and soil fauna [16,17,18]. As reported by Qian, et al. [19], there was a significant alteration in the structure of the bacterial community in MP-contaminated soil, accompanied by a notable rise in the prevalence of *Aspergillus*, *Cyanobacteria*, and *Anaplasma* species. Some species of anamorphic bacilli have the potential to cause hoof rot in domesticated ruminants [20]. The MPs were observed to induce oxidative stress effects in *Lepidium sativum*, as well as to inhibit plant growth and photosynthesis [21]. In addition, M/NPs cause mechanical damage to the external body surface of earthworms, resulting in alterations to the microbial community within the gut of earthworms and hoppers and a reduction in bacterial diversity [22,23]. From this, it can be inferred that an investigation into the impact of M/NP on soil is a crucial step in the evaluation of the ecological risk associated with microplastics.

NPs are smaller than MPs and exhibit greater mobility in soil [24]. However, the toxicological mechanism of NPs on terrestrial invertebrates remains unclear. Earthworms are large soil animals that live on the surface and play a crucial role in maintaining the functions of soil ecosystems [25]. The effects of N/MPs on soil organisms are outlined in Table 1. It has been demonstrated that earthworms may be exposed to and take up M/NPs in soil during feeding and movement [5,26]. Jiang, et al. [27] reported that 100 nm NPs are ingested by earthworms and accumulate in their bodies over time. Recent studies have confirmed that the addition of NPs to the soil environment can markedly reduce the weight of earthworms [28], resulting in oxidative stress and histopathological damage to the epidermis, intestine, and seminal vesicle of earthworms [29,30], as well as induced changes in the intestinal microbial community of earthworms [17]. Nevertheless, the potential harmful effects of NPs on earthworms remain poorly understood. Prior research has investigated the potential toxicity mechanisms of NPs through the simulation of in vivo environments, direct exposure of biological cells to contaminants, and observation of changes in intracellular biomarkers following incubation [31,32]. For example, oxidative stress, inflammation, mitochondrial fragmentation, and autophagy have been demonstrated to be pivotal events in the initial phases of NP-induced retinal damage [33]. Furthermore, NPs have been demonstrated to induce cytotoxicity and impair the function of chimeric antigen receptor T cell by altering the immune microenvironment, upregulating PD-1 and TIM-3 mRNA expression, activating the mTOR signaling pathway, and reducing cytokine release [34]. Coelomocytes are of great importance to earthworms in the recognition and removal of foreign, harmful substances. Their functions bear resemblance to those observed in vertebrate immune cells [35]. This makes the earthworm coelomocytes a suitable model for studying the toxic effects of NPs.

Polystyrene (PS) is one of the most frequently utilized plastic polymers in the manufacture of a vast array of products [40]. However, plastic products such as PS are difficult to break down in the natural environment and can also release toxic substances such as styrene monomer [41]. We investigated the uptake of PS NPs by earthworm immune cells over a 24 h period, assessed the cytotoxicity and immunotoxicity of NPs by testing cell viability, and confirmed intracellular oxidative stress by testing the viability of some representative antioxidant enzymes as well as oxidative damage indicators. The potential effects of NPs on immune cell membranes, lysosomes, mitochondria, and apoptosis were explored using lactate dehydrogenase (LDH) viability, neutral red retention time (NRRT), mitochondrial membrane potential (MMP), and apoptosis, respectively. The results of this study are expected to provide useful information for exploring the effects of NPs on terrestrial invertebrates and to facilitate the accurate assessment of the ecological risk associated with NPs.

## 2. Material and Methods

### 2.1. PS NPs

Fluorescent and nonfluorescent, monodisperse PS microspheres with a diameter of 100 nm were obtained from Zhongke Leiming (Beijing) Technology Co., Beijing, China. The suspension was sonicated with an ultrasonic device for 30 min before use. Scanning electron microscopy (SEM, Quanta 250 FEG, Hillsboro, OR, USA) was used to observe the surface morphology of the virgin PS beads, and dynamic light scattering (DLS, BI-200SM, Brookhaven, NY, USA) determined particle size distribution.

### 2.2. Earthworm Maintenance and Cell Extraction

The purchased earthworms (*E. fetida*) were acclimatized for a period exceeding 14 days in an artificial soil mixture comprising 70% industrial sand, 20% kaolin, and 10% dry cow dung. The temperature was maintained at 22.0 ± 1.0 °C, the relative humidity at 60 ± 2%, and the light–dark cycle at 12:12 h. Healthy adult earthworms (300–500 mg) were selected after a 12 h purge in the dark and washed with saline. The immune defense cells were obtained by placing the earthworms in an extraction solution (pH 7.4, adjusted with NaOH) containing 5% anhydrous ethanol, 1% guaiacol glycerol ether, 0.25% EDTA, and an appropriate quantity of saline. The cells were washed by centrifugation (4 °C, 2500 rpm, 5 min) with phosphate buffer solution (PBS) and subsequently resuspended in RPMI-1640 medium containing 1% penicillin/streptomycin and 10% fetal bovine serum. These reagents were purchased from Zhongke Maichen (Beijing) Technology Co, PS000100. The cell population was quantified using a cell counter (Countess II, MA, USA) and subsequently diluted as required for the experimental procedure [42].

### 2.3. Cellular Uptake of NPs

An appropriate quantity of earthworm immune cells was introduced to a 24-well plate (4 × 10^5^ cells/well) and, subsequently, a suspension of fluorescent NPs (at concentrations of 0, 100, 200, 300, 400, and 500 mg/L, respectively) was added in accordance with a ratio of 9:1. The cellular uptake was analyzed after an incubation period of 2 or 4 h at 37 °C without CO_2_. Following three washes with PBS (2500 rpm, 5 min), 0.2 mL was introduced to a 96-well plate and 0.05 mL of a 0.5% Triton X-100 solution (0.2 N NaOH) was added to lyse the cells. The fluorescence intensity (λex = 620 nm, λem = 680 nm) of the NPs within the requisite hole was quantified utilizing a fluorescence microplate analyzer (Genios, Tecan, Switzerland). Uptake efficiency was calculated as a percentage. Methodological references to Kulkarni and Feng [43].

### 2.4. Cytotoxicity of NPs

To ascertain the cytotoxicity of NPs in vitro, the cell counting kit-8 (CCK-8, Nanjing Jiancheng, Nanjing, China) was employed. The cells were precultured in 96-well plates for 24 h (2 × 10^5^ cells/well), and controls were medium without NPs (n = 6). The absorbance was then quantified utilizing a multifunctional microplate reader (Spark, TECAN, Männedorf, Switzerland), with the cytotoxicity of NPs calculated according to the manufacturer’s scheme.

### 2.5. Determination of Oxidative-Stress-Related Indicators

Reactive oxygen species (ROS) detection. To evaluate intracellular ROS levels after NP exposure, an appropriate amount of DCFH-DA probe was added to each treatment (1 × 10^6^ cells/well). The loading procedures were performed largely according to the manufacturer’s protocol. After incubation, the fluorescence intensity (λex = 488 nm, λem = 525 nm) of each well was measured using the fluorescent enzyme marker. A portion of the cell suspension was also taken and disrupted by sonication in an ice-water bath, which was used to obtain the total protein (TP) content. The results were expressed as fluorescence value/protein.

Superoxide dismutase (SOD) and catalase (CAT) activities and glutathione (GSH), malondialdehyde (MDA) and 8-hydroxy-2-deoxyguanosine (8-OHDG) levels. The incubated single cell suspension was centrifuged at 2500 rpm for 5 min to remove the medium and precipitated by homogenization with PBS. This step was repeated twice to remove NPs; then, the mixed solution was homogenized on ice using an ultrasonic processor (SONICS, USA) (10 × 5 s with 5 s intervals) and centrifuged. The supernatants were collected for analysis of SOD, CAT, GSH, MDA, and TP, which were purchased from Nanjing Jianjian Bioengineering Institute (Nanjing, China). The 8-OHDG test kit was purchased from Jiangsu Meibiao Biotechnology Co. (Nanjing, Jiangsu, China).

The SOD test is based on the principle that tetrazolium salts can react with superoxide radicals to form a purple product. This product has UV absorption at 450 nm; CAT activity is determined by the ammonium molybdate method, where the decomposition of H_2_O_2_ by CAT is rapidly stopped by the addition of ammonium molybdate and the remaining H_2_O_2_ interacts with the ammonium molybdate to form a yellowish complex, which is measured at 405 nm; GSH reacts with dithiobinitrobenzoic acid to form a yellow compound, which can be quantified colorimetrically at 405 nm. The determination of MDA is based on its condensation with thiobarbituric acid, a reaction that produces a red product that can be read spectrophotometrically at 532 nm. It should be noted that the mixture must be centrifuged at 4000 rpm for 10 min before testing. Intracellular 8-OHDG levels were determined by enzyme-linked immunosorbent assay (ELISA) using a two-antibody one-step sandwich assay. To the coated microtiter wells precoated with embracing antibodies, the specimen, standard, and HRP-labelled detection antibody were added sequentially, after warming and thorough washing. The color was developed with the substrate TMB, and the shade of color was positively correlated with the 8-OHDG content in the samples. The absorbance was scanned at 450 nm and the standard curve was fitted using ELISACalc software (ELISA Calc, Shanghai, China). Cell concentration and experimental design all conform to the requirements of the kit.

### 2.6. Test for the Integrity of the Cell Membrane

Cell membrane integrity was evaluated by measuring LDH activity in culture medium supernatants. For further details on cell processing methods, please refer to Section 2.5. The supernatant should then be collected and the assay performed in accordance with the instructions provided in the kit.

### 2.7. Detection of Immune-Related Indicators

Toxicity test of NP on lysosomes: The impact of NP on the stability of lysosomal membrane was evaluated through the utilization of the NRRT assay [44]. The treated earthworm immune cells (1 × 10^6^ cells/well) were collected and washed and then resuspended by the addition of an appropriate volume of PBS. The cell suspension and Ringer’s solution were combined in a 1:8 ratio, and 20 µL of the resulting mixture was deposited onto a sterile slide. The Neutral Red Working Solution was prepared in the following manner: 20 mg neutral red, 1 mL dimethyl sulfoxide, and 2.5 mL Ringer’s solution. A total of 20 μL of this solution was added to the cell mixture and immediately covered with a glass coverslip. The time required for 50% of the cells to be stained pink was recorded as NRRT under a light microscope (Nikon, Tokyo, Japan) with a magnification of 400×.

Lysozyme (LZM) activity: The procedures for handling cells, methods of homogenizing samples, and manufacturers of purchase kits were consistent with those described in Section 2.5.

Ca^2+^ levels: The cells (5 × 10^6^ cells/well) were washed on two occasions following staining, using PBS. Thereafter, an appropriate quantity of Fura-2 AM (calcium ion fluorescent probe) (Beyotime, Shanghai, China) was introduced. Following a 30 min incubation period at 25 °C, the fluorescence value of each well was determined at 335 nm and 380 nm excitation wavelengths, respectively, using a fluorescence enzyme marker. The ratio of the two was employed as an indicator of the intracellular calcium ion concentration.

### 2.8. Testing of MMP

Cellular MMP was identified through the utilization of the fluorescent probe JC-1 (Solarbio, Beijing, China). Following repeated rinsing, the requisite quantity of JC-1 probe should be added to the cells (2 × 10^6^ cells/well). After a 20 min incubation period in the absence of light, the cells were rinsed once more using JC-1 buffer. The fluorescence values of JC-1 monomer (green) at excitation and emission wavelengths of 490 nm and 530 nm were determined by means of a fluorescent enzyme labeler, while the fluorescence values of JC-1 polymer (red) were observed at 525 nm and 590 nm, respectively.

### 2.9. Apoptosis Assays

The apoptosis of cells following NP exposure was identified through the utilization of fluorescent probes, namely Annexin V-FITC and propidium iodide (PI) (Solarbio, Beijing, China). Subsequently, the stained cells (3 × 10^6^ cells/well) were taken and washed twice with PBS, before being resuspended in 500 μL of binding solution, with the cell concentration maintained at 1 × 10^5^ to 5 × 10^5^ cells per mL. Annexin V-FITC and PI were added to the cytosol at a ratio of 5 μL each, mixed thoroughly, and incubated for 15 min at room temperature in the dark. To prevent cross-talk between fluorescence channels, single-stained samples of experimental groups were prepared for fluorescence compensation. The apoptosis rate of earthworm immune cells was determined using a flow cytometer (ImageStreamX MarkII, Merck, Darmstadt, Germany) with an excitation wavelength of 488 nm, and the data were processed with FlowJo software (v10.7.2).

### 2.10. Data Analysis

The diameter of PS microspheres was analyzed using OriginPro 2022 software. Statistical analyses and graph generation were performed using SPSS Statistics 25 and GraphPad Prism 9.5, respectively. Each treatment was replicated six times and data are presented as standard error of the mean (SEM). The control was considered when the NP concentration was 0. One-way analysis of variance (ANOVA) and post hoc Tukey’s method were applied to determine statistical significance between the control and exogenous treatment groups. All experimental instruments mentioned in this paper are calibrated regularly.

## 3. Results and Discussion

### 3.1. Characterization of NPs

**Figure 1:**
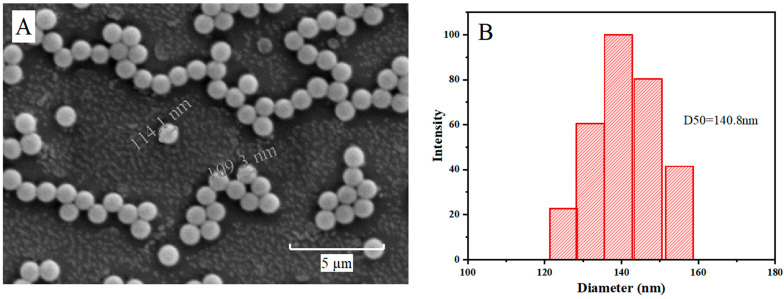
The SEM images (**A**) and particle size distribution (**B**) of 100 nm NPs.

Under SEM, the PS-NPs were observed to exhibit a regular spherical morphology with a smooth surface (Figure 1A). The hydrodynamic size of the nanoparticles dispersed in saline was determined using the DLS method (Figure 1B). The size distribution of the NP particles ranged from 124.9 to 155.1 nm, with an average diameter of 140.8 nm (the aggregation of small quantities is essentially negligible). The dimensions of the particles were observed to exceed the specifications set forth by the manufacturer, a discrepancy that may be attributed to the use of a dispersant [45].

### 3.2. The Accumulation of NPs in Cells and Their Cytotoxicity

**Figure 2:**
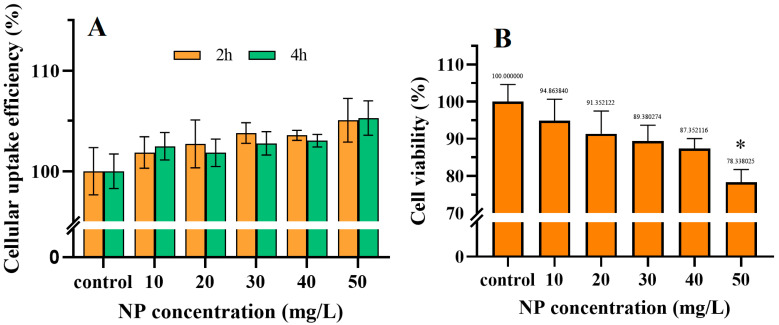
Cellular uptake efficiency measurement of *Eisenia fetida* immune cells after 2 and 4 h incubations with fluorescent NPs (**A**) and cell viability measurement after 24 h incubations with nonfluorescent NPs (**B**). Differences are considered statistically significant at * *p* < 0.05, compared with the control.

The coelomocytes of earthworms, which are present in the somatic fluid, function as immune cells and play a crucial role in eliminating exogenous substances and fighting bacterial infections [35]. The available evidence suggests that these cells exhibit a high level of phagocytic activity towards xenobiotic substances, which would render coelomocytes more susceptible to NP [46,47]. By means of confocal z-stack imaging, Roshanzadeh, et al. [48] discovered that the majority of 200 nm NPs were capable of attaching to the plasma membrane of neonatal rat ventricular myocytes within five minutes and subsequently transferring to the intracellular space over time. To ascertain the uptake efficiency of 100 nm PS, the fluorescence intensity of earthworm coelomocytes was evaluated following a 2 and 4 h co-incubation with fluorescent NPs. The data were expressed as a percentage relative to the control (Figure 2A). The results demonstrated that higher concentrations may facilitate a greater cellular uptake efficiency, while the incubation time had no significant impact on cellular uptake. Similar to our results, significant uptake of plastic particles was observed in HT29-MTX cells after 24 h exposure to 100 mg/L NPs [49].

In order to ascertain the cytotoxicity of NPs, it became necessary to evaluate the viability of earthworm immune cells, which was achieved through the utilization of CCK-8. As illustrated in Figure 2B, the data demonstrate a clear dose–effect relationship, with cell viability in the 50 mg/L treatment group being 78.3% of that in the control group. This indicates that higher concentrations of NPs have a greater impact on cell survival. This finding is consistent with previous internalization experiments. Additionally, the toxic effects of PS on other immune cells, including the human microglial cells (HMC-3) [50] and the human macrophages (THP-1) [51], were also observed. To gain insight into the underlying mechanisms responsible for the observed decline in cell viability, a comprehensive examination of the cellular responses was conducted.

### 3.3. The Oxidative Stress Induced by NPs

**Figure 3:**
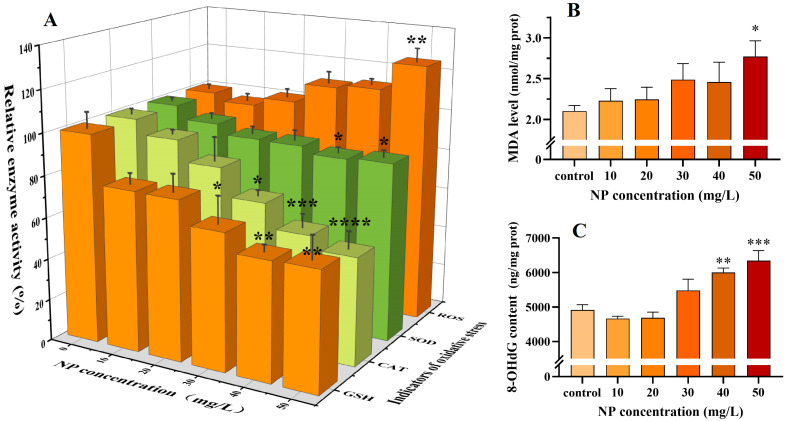
Changes in oxidative-stress-related indices of ROS, SOD, CAT, and GSH (**A**), MDA (**B**), and 8-OHdG (**C**) in *Eisenia fetida* immune cells after exposure to NPs for 24 h. A statistically significant difference is deemed to exist at * *p* < 0.05, ** *p* < 0.01, *** *p* < 0.001, and **** *p* < 0.0001, in comparison with the control.

ROS are by-products of aerobic cellular respiration that possess high chemical reactivity. In physiological conditions, moderate levels of ROS can facilitate immune responses, promote cell growth and repair, and protect cells from pathogens. However, elevated levels of ROS frequently indicate cellular damage and disrupted cellular homeostasis [52]. Prior research has demonstrated that NPs can affect the viability of immune cells through a range of mechanisms associated with oxidative stress induction, including necrosis and apoptosis [53]. It can thus be hypothesized that the observed decrease in cell viability may be attributable to NP-mediated oxidative stress. The levels of ROS in the treated cells were gauged using the DCFH-DA method, as illustrated in Figure 3A. The findings indicated that, at 10 mg/L, the intracellular ROS level exhibited a slight decline, followed by a gradual increase, reaching a markedly higher level than that observed for the control at 50 mg/L. This demonstrated that the redox balance of the cells was disrupted and the elevated ROS levels may have compromised the earthworms’ defense system, resulting in cellular toxicity and mortality under oxidative stress [54].

To safeguard earthworm immune cells from oxidative stress, the antioxidant defense system will be initiated [55]. Among the enzymes, SOD and CAT are particularly noteworthy representatives of the cellular antioxidant system. SOD catalyzes the disproportionation of O_2_^−^• to produce O_2_ and H_2_O_2_, while CAT decomposes H_2_O_2_ into H_2_O and O_2_ [16,56]. These two enzymes are functionally interrelated and work together to regulate cell signaling. GSH is a vital nonenzymatic antioxidant that safeguards cells from oxidative damage by neutralizing ROS and certain pro-oxidants [57]. The combined action of these three antioxidants provides protection for earthworms from oxidative stress and reduces the risk of ROS and lipid peroxidation [58]. The current study revealed a notable correlation between the trends of SOD, CAT, and GSH (Figure 3A). A decreasing trend was observed in all NP-treated groups, with the lowest levels of SOD and GSH, at 40 mg/L, representing 86.2% and 22.8% of the control, respectively. CAT activity decreased to 51.7% of the control at 50 mg/L. The observed decline can be attributed to the fact that, during acute exposure, cells are subjected to considerable stress, resulting in the overproduction of ROS that overwhelms the cellular antioxidant defense system [59,60]. ROS that are insufficiently promptly consumed may attack the active sites of enzyme proteins, resulting in a significant depletion of SOD and CAT [61]. Furthermore, direct interactions between NPs and enzyme proteins may also be responsible for the observed decrease in enzyme viability. As documented by Wang, et al. [62] and Hu, et al. [63] using spectroscopic means, NPs are capable of disrupting the structure of SOD and CAT, which, in turn, affects enzyme function. PS has been demonstrated to be capable of conjugating with GSH, which may also be a contributing factor in GSH depletion [59]. The observed alterations in SOD and CAT activities, as well as GSH levels, suggest that the antioxidant capacity of earthworm immune cells is compromised.

Additionally, elevated levels of ROS may oxidize cellular lipids, including polyunsaturated fatty acids and phospholipids [64]. The resulting MDA is a by-product of this process and has been demonstrated to damage biomolecules such as proteins, nucleic acids, and other biomolecules within the cell, thereby contributing to cellular ageing and lesions [65]. In this study, MDA levels were found to be elevated in all treatment groups relative to the control. A statistically significant difference (*p* < 0.05) was noted at 50 mg/L (Figure 3B). These findings indicate that NP treatment induces oxidative stress and lipid peroxidation damage in the immune cells of earthworms.

Furthermore, cellular DNA is also highly susceptible to attack by ROS [66]. 8-OHDG content is a typical biomarker reflecting oxidative damage to DNA [67]. The intracellular levels of 8-OHDG were examined using an enzyme immunoassay, which revealed no significant change in the 8-OHDG content of the low NP-treated groups (10, 20, and 30 mg/L). However, 8-OHDG was significantly elevated in the high-dose NP-treated groups (40 and 50 mg/L). The trends of 8-OHDG and MDA (Figure 3B) were similar to those of ROS (Figure 3A). This indicates that NP exposure will result in irreversible oxidative damage to cells, with a discernible dose–response relationship.

### 3.4. NPs Without Effect on Cell Membranes

**Figure 4:**
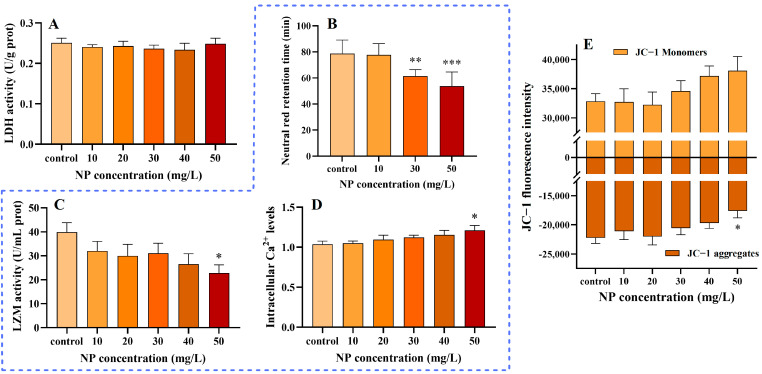
Impact of NP exposure on LDH activity (**A**), NRRT (**B**), LZM activity (**C**), Ca^2+^ level (**D**), and fluorescence intensities of JC-1 (**E**) in *Eisenia fetida* immune cells after 24 h of treatment. Differences are considered statistically significant at * *p* < 0.05, ** *p* < 0.01, and *** *p* < 0.001, compared with the control.

The plasma membrane represents the primary barrier that hinders the unhindered entry and exit of xenobiotics into and out of the organism. It plays a pivotal role in maintaining intracellular homeostasis [68]. It was demonstrated that, upon approaching the plasma membrane, the -OH groups on the surface of PS exhibited a pronounced affinity for the R-N(CH_3_)^3+^ or PO_4_^−^ groups present in phospholipids [69]. Once a threshold level of plastic particles has been adsorbed onto the membrane, the membrane may rupture or cells may leak. In healthy cells, LDH is a soluble protein that is discharged from the cell when the plasma membrane is compromised. As illustrated in Figure 4A, there was no notable alteration in the LDH concentration within the cell culture supernatant at the experimental concentrations. This indicates that plasma membrane damage may not be the primary mechanism of cell death and that PS in close proximity to cells may be actively phagocytosed by earthworm immune cells [47].

### 3.5. Influence of NPs on the Immune System

The aforementioned data have demonstrated the accumulation of particles and oxidative stress in earthworm immune cells following exposure to NPs. Nevertheless, the impact of NPs on the earthworm immune system remains largely unknown. Lysosomes are of pivotal importance in the field of cellular immunity, modulating the immune response by degrading and digesting invading foreign substances and damaged cells and activating antigen presentation processes [70]. Previous studies have demonstrated that PS is internalized into cells primarily through lysosomes [71,72]. Neutral red is a weak cationic dye that can freely enter living cells and accumulate in organelles like mitochondria and lysosomes [73]. Accordingly, the impact of NP exposure on lysosomal functionality was evaluated through the detection of NRRT. It was observed that the NRRT of the cells exhibited a significant reduction with increasing dose, demonstrating a clear dose-dependent trend (Figure 4B). The shortest NRRT was observed in the NP treatment group (50 mg/L) at a duration of 53.67 min. These findings indicate that exposure to NPs may exert an influence on the lysosomes of earthworm immune cells. This may be attributed to the accumulation of NPs in lysosomes, which may result in the destabilization or rupture of the lysosomal membrane, thereby impeding autophagy and cell clearance processes. Ultimately, this could lead to the demise of immune cells [45,74].

The release of LZM by earthworm immune cells represents the most prevalent innate immune response to pathogens and parasites [75]. LZM possesses the capacity to cleave polysaccharide chains within the cell walls of bacteria, leading to their destruction or the inhibition of their growth [76]. Furthermore, LZM has the capacity to modulate the response of the immune system, activating macrophages and other immune cells and promoting their production of more inflammatory mediators and cytokines. This enhances the body’s resistance to infection [77]. LZM expression has been demonstrated to be upregulated in *Hypophthalmichthys molitrix* [78], *Mytilus* [79], and *Bombyx mori* [80] following exposure to PS. Consequently, we evaluated the cytotoxicity and immunotoxicity induced by NP exposure by assessing LZM activity in earthworm immune cells, with the results presented in Figure 4C. At 50 mg/L, the bioactivity of LZM was observed to be 22.8 U/mg prot, exhibiting a notable decline in comparison to the control group, which demonstrated a value of 39.8 U/mg prot, representing a decrease of 42.7%. Our findings indicate that the stimulation of NPs inhibited the bioactivity of intracellular LZM. A reduction in LZM may result in diminished immune cell resistance to microorganisms and an elevated risk of bacterial infection [81].

Ca^2+^ is primarily stored in the endoplasmic reticulum of cells and plays a pivotal role in the innate immunity of earthworms [82]. Furthermore, Ca^2+^ signaling has been demonstrated to regulate phagocytosis and the antioxidant defense system in earthworm immune cells in response to environmental stresses and pathogen invasion. Figure 4D illustrates the Ca^2^⁺ levels of earthworm immune cells subjected to NPs exposure. It demonstrates an upregulation trend in the intracellular Ca^2^⁺ levels, with a 17.5% elevation at 50 mg/L compared to the control. Our findings provide further evidence to support the conclusions of previous studies, which have elucidated the impact of NP exposure on the immune system of earthworms. This is evidenced by damage to lysosomes, inhibition of LZM viability, and impact on intracellular calcium homeostasis.

### 3.6. Impact of NPs on MMPs

Mitochondria are the ’power plants’ of the cell and are also associated with the regulation of the redox state, cell signaling, and ion homeostasis. In the respiratory chain of mitochondria, the energy released during electron transfer is utilized to facilitate the pumping of protons within the matrix into the membrane gap. This results in an internally negative and externally positive potential difference, or MMP [83]. Normal MMP is essential for the maintenance of cellular physiological functions. Consequently, the alterations in the intracellular MMP following exposure to varying concentrations of NPs for a period of 24 h were monitored using the JC-1 probe. In cells with normal MMP, JC-1 was observed to aggregate in the mitochondrial matrix, producing red fluorescence. In cells that have undergone damage, the MMP is observed to become low, with JC-1 tending to exist as a monomer and emitting green fluorescence. The shift from a high to a low MMP is regarded as an early marker of apoptosis [84]. During the experiments, an increase in the fluorescence intensity of the JC-1 monomer was observed, whereas that of the polymer decreased and exhibited a statistically significant difference when compared to the control at 50 mg/L (Figure 4E). This alteration indicates a reduction in MMP and an impairment in mitochondrial function. Cao, et al. [85] observed in a cadmium-induced apoptosis assay in BEAS-2B cells that cadmium induces cellular oxidative stress, which results in the accumulation of ROS, the activation of the MAPK signaling pathway, and, ultimately, the activation of the mitochondria-mediated apoptotic pathway.

### 3.7. Apoptosis Induced by NPs

**Figure 5:**
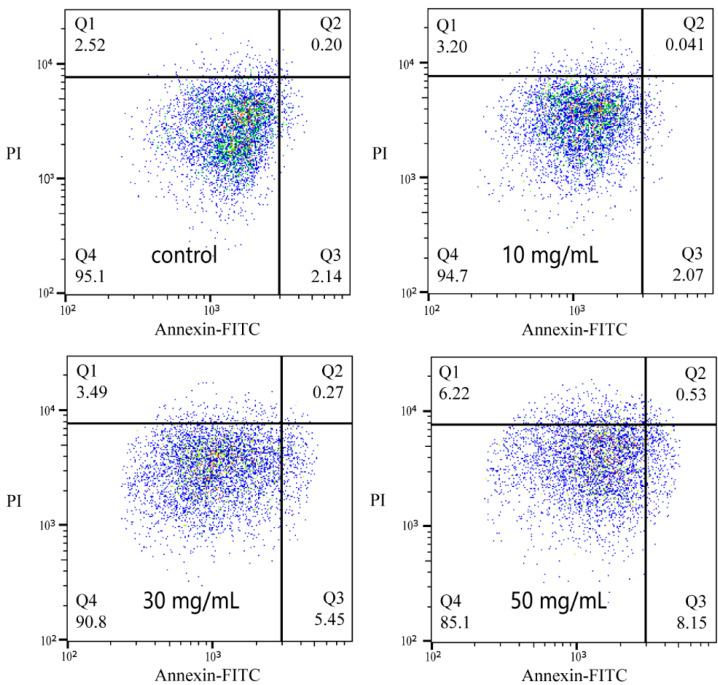
Induction of apoptosis by PS NP exposure in *Eisenia fetida* immune cells after 24 h of treatment.

To further validate the conclusions presented in Section 3.6, the cells were double-stained using Annexin V-FITC and PI probes, and the rate of apoptosis was assessed by flow cytometry. The images are presented in Figure 5, wherein the Q1, Q2, Q3, and Q4 regions represent necrotic cells, late apoptotic cells, early apoptotic cells, and normal cells, respectively. As the concentration of NP exposure increased, the number of cells in a normal state decreased, while the number of cells exhibiting early apoptosis, late apoptosis, and necrosis increased. The induction of early apoptosis and cell necrosis was particularly pronounced. Compared to the control group, the proportion of cells exhibiting early apoptosis increased by a factor of 2.81, while the proportion of necrotic cells increased by a factor of 1.47 at the maximum exposure concentration of NP. These findings indicate that NP can induce apoptosis and necrosis in earthworm immune cells. This finding is consistent with previous studies in which PS NP has been demonstrated to induce apoptosis in normal cells, such as mouse splenic lymphocytes and human trophoblast cells [84,86].

## 4. Conclusions

This study demonstrated that 100 nm polystyrene NPs can be internalized by earthworm immune cells and caused a 21.7% (50 mg/L) decrease in cell viability after 24 h. Mechanistic analysis revealed that NPs increased ROS production, decreased antioxidant activities (e.g., SOD, CAT, and GSH), and caused oxidative damage, resulting in increased intracellular MDA and 8-OHdG levels by 31.8% and 29.1%, respectively. In addition, NPs shortened the NRRT to 53.67 min, significantly reduced the activity of the immunoprotein LZM, and increased intracellular Ca^2^⁺ levels to 116.8% of the control. NPs also reduced MMP, triggering apoptosis without significantly disrupting plasma membranes.

The interconnected effects of oxidative stress, immune responses, and apoptosis highlight the complex mechanisms of NP toxicity. These findings provide valuable insights into NPs’ ecological risks to soil fauna, emphasizing the need for further research to evaluate their broader environmental impacts.

## Figures and Tables

**Table 1:** Summary of studies investigating the impact of M/NPs on soil organisms.

Polymer Type	Size, μm	Soil Biology	Major Findings	Reference
Polystyrene	0.1, 10	*Eisenia fetida*	The M/NPs disrupted the integrity of the earthworm intestine and promoted the accumulation of pyrene by the earthworms.	[36]
Polystyrene	0.2	*Eisenia fetida*	NPs exposure provoked oxidative stress, neurotoxicity, and developmental and reproductive toxicity.	[37]
Polyethylene	200~300	*Eisenia fetida*	MPs cause weight loss, growth inhibition, and death in earthworms	[38]
Polypropylene (PP), polyethylene (PE), polyvinylchloride (PVC)	0~125	Garden cress (*L. sativum*)	In the chronic exposure experiment (21 days), PP and PE exerted a detrimental effect on the germination rate, number of leaves, and biomass of lettuce, while its height was predominantly influenced by PE + PVC.	[21]
Polystyrene	0~500	springtail (*Folsomia candida)*	The MPs altered the microbial community in the gut of the springtails, inhibiting their reproduction and causing them to exhibit avoidance behaviors.	[22]
Polystyrene	0.02, 0.1	Bacterium (*Rhodococcus jostii*)	NPs have the capacity to inhibit the transformation of tetrabromobisphenol A by the Gram-positive bacterium *Rhodococcus jostii*.	[39]
Polyethylene	180, 250	*Eisenia fetida*	MPs caused damage to the male reproductive organs of earthworms, whereas the effect on the female reproductive organs was negligible.	[30]

## Data Availability

Data will be made available on request.
